# Seizure-induced LIN28A disrupts pattern separation via aberrant hippocampal neurogenesis

**DOI:** 10.1172/jci.insight.175627

**Published:** 2024-01-09

**Authors:** In-Young Choi, Jung-Ho Cha, Seong Yun Kim, Jenny Hsieh, Kyung-Ok Cho

**Affiliations:** 1 Department of Pharmacology, College of Medicine,; 2 Department of Anatomy, College of Medicine,; 3 Department of Biomedicine & Health Sciences, and; 4 Catholic Neuroscience Institute, The Catholic University of Korea, Seoul, Republic of Korea.; 5 Department of Neuroscience, Developmental and Regenerative Biology, and; 6 Brain Health Consortium, The University of Texas at San Antonio, San Antonio, Texas, USA.; 7 Institute for Aging and Metabolic Diseases and; 8 CMC Institute for Basic Medical Science, the Catholic Medical Center of The Catholic University of Korea, Seoul, Republic of Korea.

**Keywords:** Neuroscience, Stem cells, Adult stem cells, Epilepsy, Memory

## Abstract

Prolonged seizures can disrupt stem cell behavior in the adult hippocampus, an important brain structure for spatial memory. Here, using a mouse model of pilocarpine-induced status epilepticus (SE), we characterized spatiotemporal expression of *Lin28a* mRNA and proteins after SE. Unlike *Lin28a* transcripts, induction of LIN28A protein after SE was detected mainly in the subgranular zone, where immunoreactivity was found in progenitors, neuroblasts, and immature and mature granule neurons. To investigate roles of LIN28A in epilepsy, we generated *Nestin-Cre:Lin28a^loxP/loxP^* (conditional KO [cKO]) and *Nestin-Cre:Lin28a^+/+^* (WT) mice to block LIN28A upregulation in all neuronal lineages after acute seizure. Adult-generated neuron- and hippocampus-associated cognitive impairments were absent in epileptic LIN28A-cKO mice, as evaluated by pattern separation and contextual fear conditioning tests, respectively, while sham-manipulated WT and cKO animals showed comparable memory function. Moreover, numbers of hilar PROX1-expressing ectopic granule cells (EGCs), together with PROX1^+^/NEUN^+^ mature EGCs, were significantly reduced in epileptic cKO mice. Transcriptomics analysis and IHC validation at 3 days after pilocarpine administration provided potential LIN28A downstream targets such as serotonin receptor 4. Collectively, our findings indicate that LIN28A is a potentially novel target for regulation of newborn neuron-associated memory dysfunction in epilepsy by modulating seizure-induced aberrant neurogenesis.

## Abstract 2

<p>Deletion of LIN28A in all neuronal lineages alleviates both hippocampal and pattern separation memory impairments in chronic epilepsy by mitigating aberrant hippocampal neurogenesis.</p>

## Graphical abstract

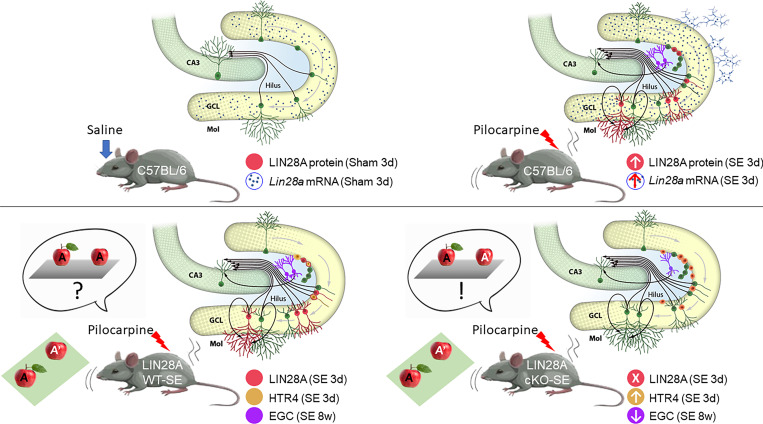

## Introduction

Contextual learning, spatial memory, and pattern separation (PS) are intricate cognitive processes involving hippocampal function and, to a certain extent, adult neurogenesis (1, 2). In the adult hippocampus, neural stem cells constantly give rise to new neurons, which later are integrated into the dentate gyrus (DG) (3–5). However, a wide variety of brain insults, including epileptic seizures, can disrupt these neural circuits, contributing to cognitive impairment in chronic epilepsy (6–8). Interestingly, repetitive, abnormal neural activity can cause aberrant hippocampal neurogenesis, represented by histologic features such as increased proliferation of neural progenitors, persistent hilar basal dendrites, and the production of mismigrated hilar ectopic granule cells (EGCs) (9). When this hippocampal neurogenesis is inhibited by genetic ablation or pharmacological approaches, epilepsy-associated cognitive decline is normalized successfully (10–12), suggesting that targeting seizure-generated abnormal neurons could modulate hippocampal memory dysfunction under pathological conditions. However, despite intensive efforts to elucidate the molecular mechanisms that underlie abnormal hippocampal neurogenesis after acute seizure activity, critical factors regulating seizure-generated neurons remain to be understood.

LIN28A is an RNA-binding protein that is highly expressed in stem cells (13, 14). We have previously shown that, during development, LIN28A can promote the proliferation of neural progenitors (15) as well as the survival and maturation of newborn neurons (16, 17); these are findings supported by other groups (18, 19). Moreover, LIN28A has versatile roles in various biological processes, including glucose metabolism (20, 21), tissue regeneration (22), regulation of body size (18, 20), and cancer progression (23). In relation to diseases of the central nervous system, LIN28A expression was reported to increase in glial cells after spinal cord and retinal injuries (24, 25). Given the role of LIN28A during embryonic cortical neurogenesis, it is plausible that LIN28A can play an important role in seizure-induced abnormal neurogenesis and hippocampal memory function.

Therefore, in the present study, we first assessed the spatiotemporal expression patterns of *Lin28a* mRNA and protein after pilocarpine-induced status epilepticus (SE); we then performed phenotypic analysis of LIN28A induction after acute seizure. Because multiple cell types in DG such as neural progenitors, neuroblasts, and immature and mature granule neurons expressed LIN28A protein, we generated *Nestin-Cre:Lin28a^loxP/loxP^* (LIN28A conditional KO [cKO]) mice to block LIN28A induction in all the neuronal lineages after acute seizures. We found that LIN28A-cKO mice after SE showed alleviation of memory impairments as assessed by PS and contextual fear conditioning (CFC) tests, which are specific tests for newborn neurons and hippocampal function, respectively. However, sham-manipulated *Nestin-Cre:Lin28a^+/+^* (LIN28A WT) and LIN28A-cKO mice demonstrated no difference in PS and CFC tests, suggesting a specific role of LIN28A in epilepsy. We further demonstrated that the LIN28A-cKO mice showed a significant reduction in the number of hilar EGCs — more specifically, PROX1/NEUN-expressing mature EGCs — in the epileptic DG. Additionally, transcriptomics analysis at 3 days after acute seizure revealed a difference in gene expression pattern between LIN28A WT and LIN28A-cKO mice, providing serotonin signaling molecules as potential molecular targets of LIN28A. Finally, we confirmed that the immunoreactivity to serotonin receptor 4 (HTR4) was colocalized in LIN28A-expressing cells, suggesting HTR4 as a potential LIN28A-downstream target in epilepsy.

## Results

### Spatiotemporal pattern of Lin28a transcription and its cellular phenotype after acute seizure.

**Figure 1:**
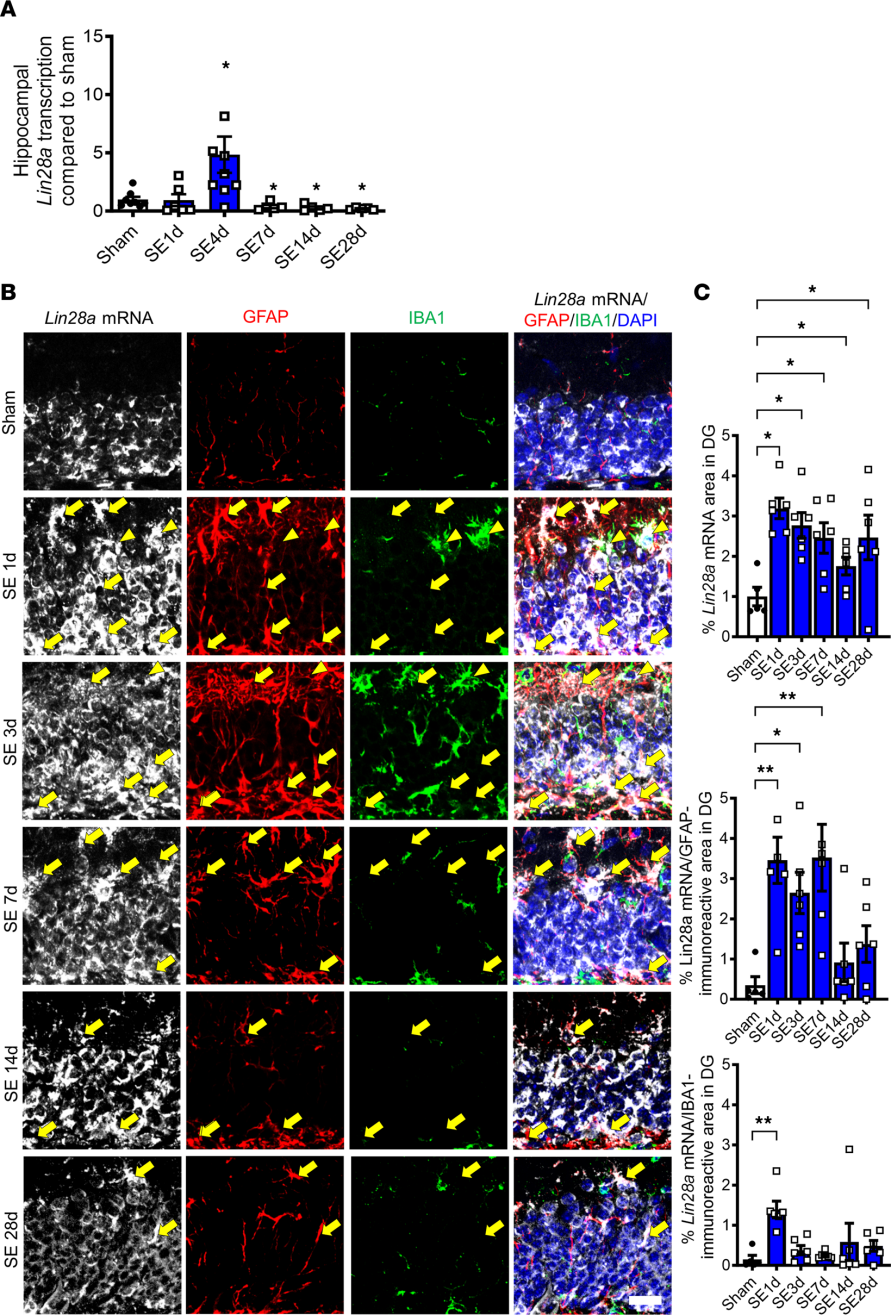
Spatiotemporal expression and cellular phenotypes of *Lin28a* transcripts in the hippocampus after pilocarpine-induced status epilepticus (SE). (**A**) A graph showing hippocampal *Lin28a* mRNA expression after SE. Mann-Whitney *U* test compared the experimental and sham groups. Sham vs. SE 1 day (d): *P =* 0.345, *U* = 16.000; sham vs. SE 4 d: *P* = 0.015, *U* = 11.000; sham vs. SE 7 d: *P* = 0.045, *U* = 6.000; sham vs. SE 14 d: *P* = 0.019, *U* = 4.000; sham vs. SE 28 d: *P* = 0.005, *U* < 0.001. Sham (*n* = 8), SE 1 d (*n* = 6), SE 4 d (*n* = 9), SE 7 d (*n* = 5), SE 14 d (*n* = 5), SE 28 d (*n* = 5). (**B**) Triple labeling of *Lin28a* mRNA and GFAP and IBA1 proteins after pilocarpine-induced SE. In situ hybridization with *Lin28a* antisense probe showed increased *Lin28a* transcripts at 1 d after SE, which was maintained for 28 d. Small cells showing *Lin28a* mRNA signals (white) were colocalized with GFAP (red) and IBA1 (green) immunoreactivity, indicating a mixture of astrocytes (yellow arrows) and microglia (yellow arrowheads). Scale bar: 200 μm. (**C**) Graphs showing temporal *Lin28a* mRNA, *Lin28a*/GFAP, and *Lin28a*/IBA1 in DG. Top graph: Welch’s ANOVA, *P* = 0.001, W(5.000, 13.410) = 8.184. Middle graph: Kruskal-Wallis *H* test, *P =* 0.002, *H* = 18.630. Bottom graph: Kruskal-Wallis *H* test, *P =* 0.007, *H* = 15.710. All tests were followed by 2-stage linear step-up procedure of Benjamini, Krieger, and Yekutieli post hoc test. Sham (*n* = 5), SE 1 d (*n* = 6), SE 3 d (*n* = 6), SE 7 d (*n* = 6), SE 14 d (*n* = 6), SE 28 d (*n* = 6). Data are presented as mean ± SEM. *P < 0.05, **P < 0.01.

To examine the spatiotemporal expression pattern of *Lin28a* mRNA after pilocarpine-induced SE, quantitative PCR (qPCR) and in situ hybridization were performed. Quantitative analysis of hippocampal *Lin28a* expression by qPCR demonstrated that hippocampal *Lin28a* transcription was significantly increased at 4 days after acute seizures (Figure 1A), indicating seizure-induced alteration of *Lin28a* transcription. We further examined temporal *Lin28a* transcription by in situ hybridization and found that a digoxigenin-labeled (DIG-labeled) antisense probe to *Lin28a* revealed specific *Lin28a* signals in DG after acute seizure (Figure 1B), whereas hybridization with a sense probe did not show any cellular labeling (data not shown). Specifically, compared with sham-controls, the signal for *Lin28a* mRNA in DG was significantly increased at 1 day after SE and was maintained until 28 days after SE (Figure 1, B and C). Interestingly, in addition to the signal for *Lin28a* mRNA in granule cell layer (GCL), additional labeling was observed after SE in the molecular layer and the hilus of DG (Figure 1B). When we used a combination of in situ hybridization and IHC to determine cell types expressing *Lin28a* mRNA after acute seizures (Figure 1, B and C), double labeling of *Lin28a* mRNA and GFAP or IBA1 proteins was observed, indicating that *Lin28a*-expressing cells were either GFAP-expressing astrocytes or IBA1-expressing microglia. Quantitative analysis of double-positive area demonstrated that *Lin28a* mRNA–expressing astrocytes were significantly increased from 1 day to 7 days after pilocarpine injection, whereas *Lin28a* mRNA–expressing microglia were increased at 1 day after SE (Figure 1C). Collectively, our findings demonstrate neuronal and glial upregulation of *Lin28a* transcription in DG after pilocarpine-induced SE.

### Seizure-induced LIN28A protein expression pattern and its cellular phenotypes in the DG.

**Figure 2:**
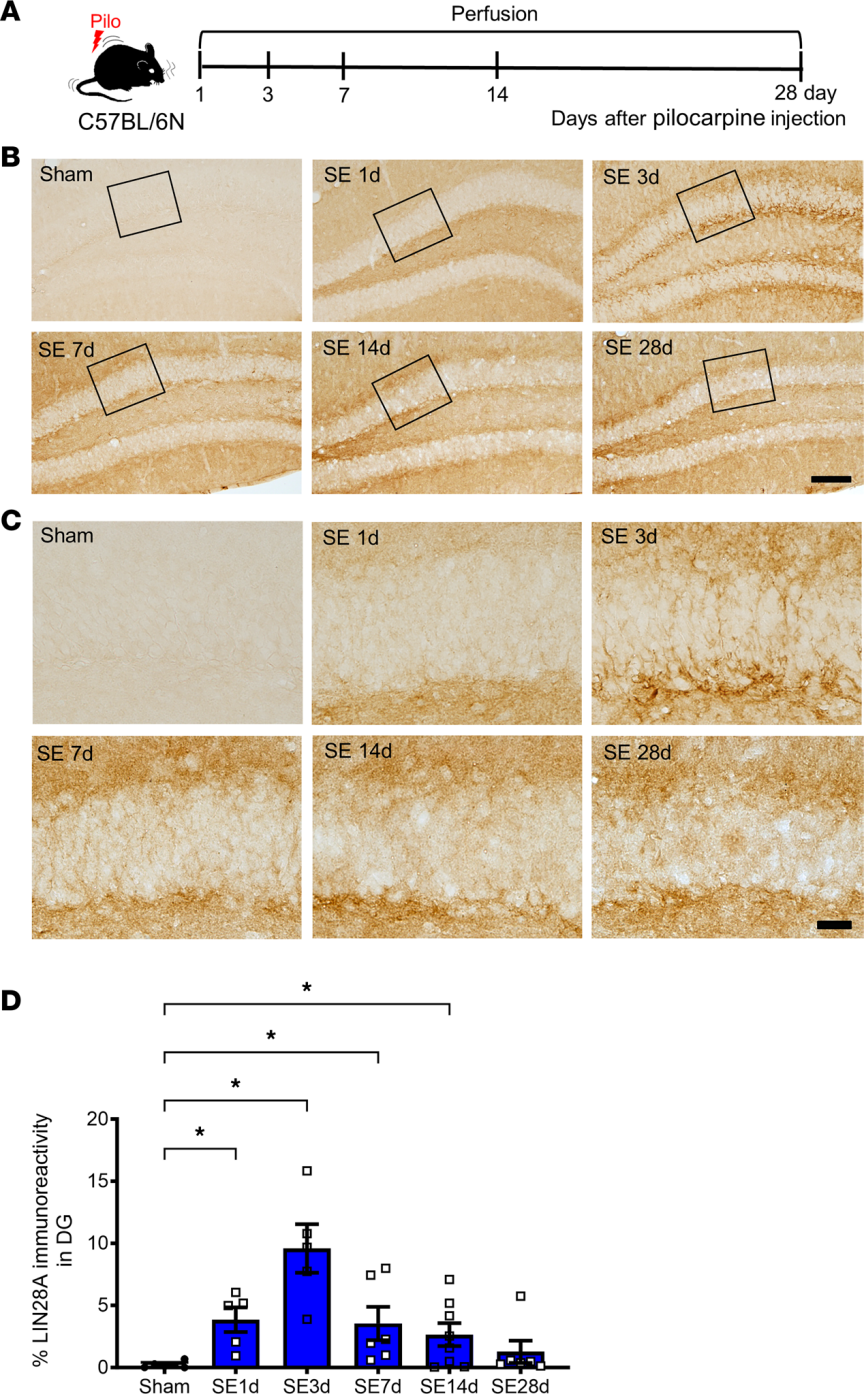
Spatiotemporal expression of LIN28A protein in the hippocampus after pilocarpine-induced status epilepticus (SE). (**A**) Experimental timeline. (**B**) LIN28A immunoreactivity in the dentate gyrus (DG) was examined. One day after acute seizures, the LIN28A signal started to be induced in the subgranular zone (SGZ) compared with that in the sham control. From 3 d to 14 d after SE, LIN28A expression was increased in the SGZ of the dentate gyrus and then decreased gradually 14 d after acute seizures. Scale bar: 50 μm. (**C**) Highly magnified images from **B** of LIN28A immunoreactivity in the dentate gyrus. After acute seizures, LIN28A expression was found mainly in the SGZ in addition to the granule cell layer and inner molecular layer of the dentate gyrus. Scale bar: 20 μm. (**D**) A graph showing the percentage of LIN28A immunoreactivity in DG after acute seizure. Kruskal-Wallis *H* test with 2-stage linear step-up procedure of Benjamini, Krieger, and Yekutieli post hoc test was performed. *P =* 0.001, *H* = 19.760. Sham (*n* = 6), SE 1 d (*n* = 5), SE 3 d (*n* = 5), SE 7 d (*n* = 6), SE 14 d (*n* = 8), SE 28 d (*n* = 6). Data are presented as mean ± SEM. *P < 0.05.

**Figure 3:**
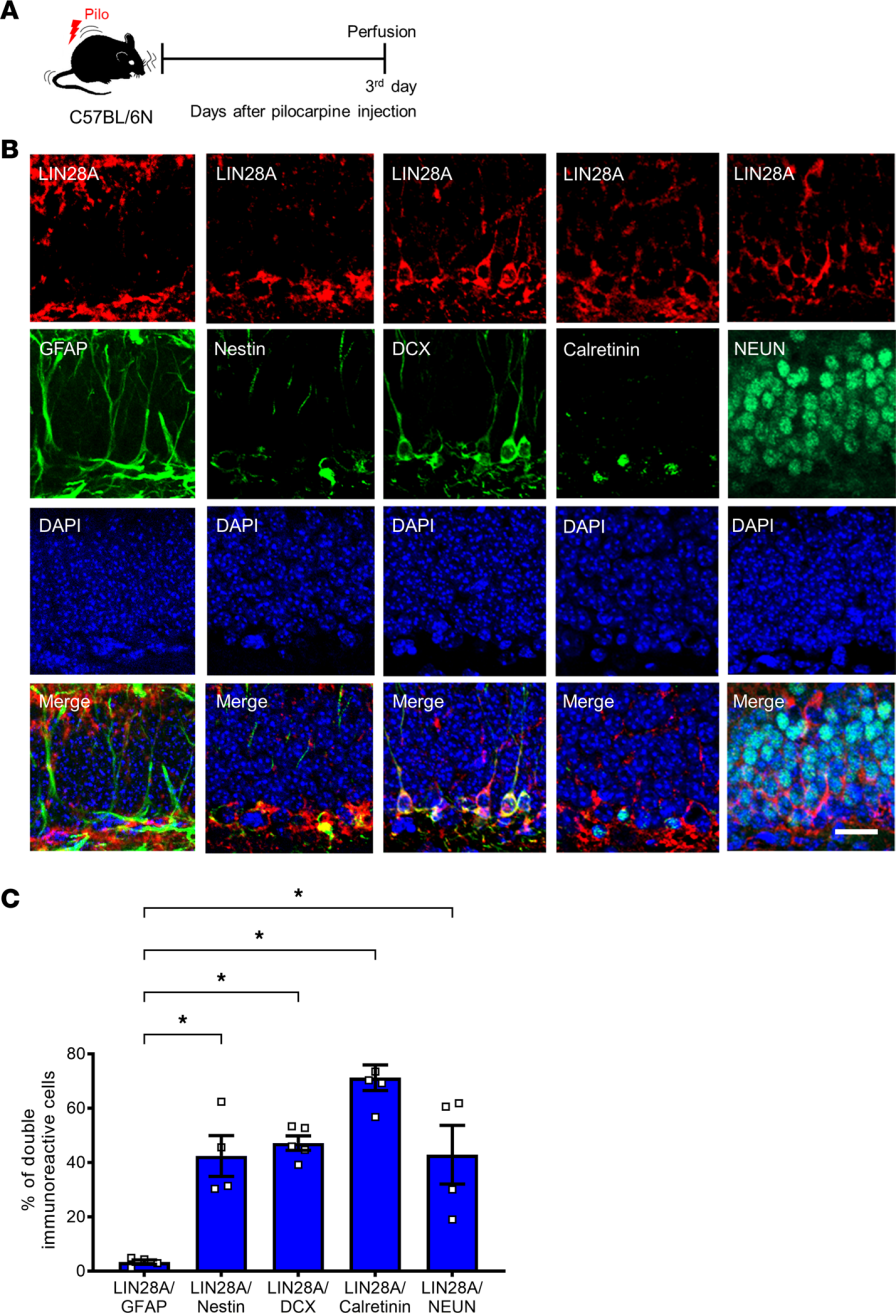
Cellular phenotypes of LIN28A expression in the subgranular zone (SGZ) of the dentate gyrus after pilocarpine-induced status epilepticus (SE). (**A**) Experimental timeline. (**B**) Double immunofluorescence of LIN28A and stage-specific markers of hippocampal neurogenesis at 3 d after SE showed that LIN28A immunoreactivity colocalized with Nestin, DCX, calretinin, and NEUN, indicating type 2 progenitors, neuroblasts, immature, and mature granule neurons. However, LIN28A expression in the SGZ did not colocalize with GFAP, suggesting that type 1 neural stem cells did not express LIN28A. Scale bar: 20 μm. (**C**) A graph showing the phenotypic analysis of LIN28A-expressing cells after acute seizure. Welch’s ANOVA was performed, followed by 2-stage linear step-up procedure of Benjamini, Krieger, and Yekutieli post hoc test. *P* = 0.001, W(4.000, 7.210) = 89.650. GFAP^+^/LIN28^+^ (*n* = 4), Nestin^+^/LIN28^+^ (*n* = 4), DCX^+^/LIN28^+^ (*n* = 5), calretinin^+^/LIN28^+^ (*n* = 5), NEUN^+^/LIN28^+^ (*n* = 4). Data are presented as mean ± SEM. *P < 0.05.

Next, we investigated the spatiotemporal expression of LIN28A protein after pilocarpine-induced SE (Figure 2A). Compared with the sham controls, which showed no apparent LIN28A immunoreactivity in DG, LIN28A-expressing cells started to appear in DG from 1 day after pilocarpine treatment (Figure 2, B and C). Immunoreactivity to LIN28A peaked at 3 days after SE and then gradually declined beginning at 7 days after SE. Quantitative analysis of LIN28A immunoreactivity clearly showed that LIN28A expression was significantly upregulated from 1 day to 14 days after SE (Figure 2D), showing the induction of LIN28A after seizure activity. Because LIN28A protein expression was detected mainly in the subgranular zone (SGZ) of DG after pilocarpine-induced SE (Figure 2, B and C), we next assessed the cellular phenotype of LIN28A-expressing cells using double immunofluorescence with stage-specific markers for hippocampal neurogenesis (Figure 3A). Immunolabeling for LIN28A at 3 days after SE revealed that this protein colocalized with nestin, doublecortin (DCX), calretinin, and neuronal nuclei (NEUN), which are markers for neural stem/progenitors, neuroblasts, and immature and mature granule neurons, respectively (Figure 3B). Moreover, LIN28A labeling did not overlap with GFAP^+^ cells, a marker for type 1 neural stem cells, suggesting that LIN28A was expressed in type 2 progenitors but not in hippocampal stem cells (Figure 3B). Quantitative analysis of the percentage of double-immunoreactive cells showed that LIN28A immunoreactivity was observed in various cell types from neural progenitors to mature neurons, revealing that the most frequent cell type expressing LIN28A was the calretinin^+^ immature granule cell (Figure 3C). Taken together, these results demonstrate that acute seizures can induce LIN28A protein expression in subgranular cells of multiple neuronal lineages during the early postseizure period.

### Mice with LIN28A deletion in all neuronal lineages show attenuation of PS and spatial contextual memory deficits after pilocarpine-induced SE.

**Figure 4:**
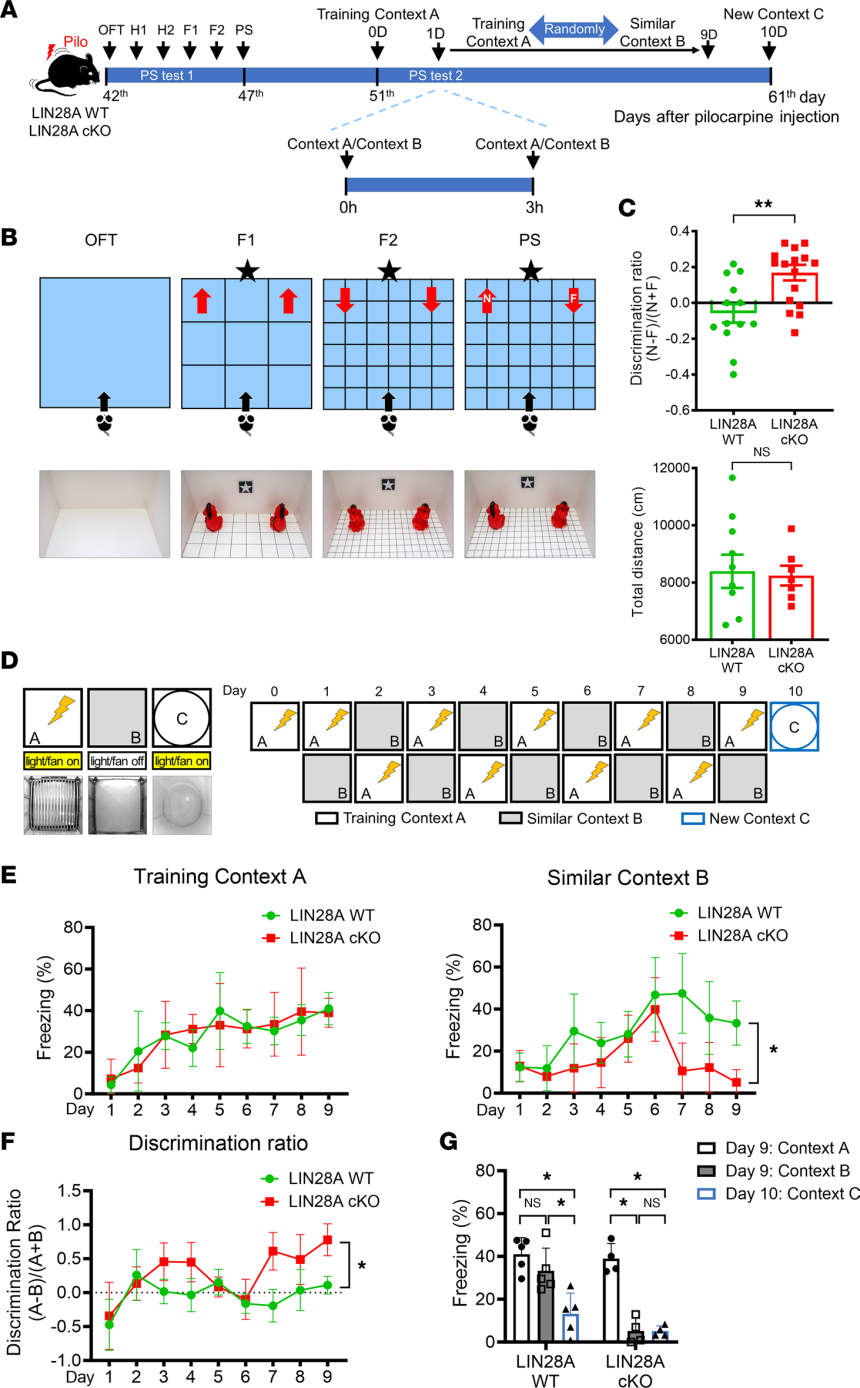
Amelioration of pattern separation (PS) memory impairment in epileptic LIN28A-cKO mice. (**A**) Experimental timeline. (**B**) A schematic of OFT and PS test 1. (**C**) Graphs showing discrimination ratio and distance moved. LIN28A-cKO mice significantly improved their novel object recognition. Student’s *t* test was performed. Discrimination ratio: *P* = 0.002, *t*(28) = 3.364. WT (*n* = 13), cKO (*n* = 17); distance moved: *P* = 0.848, *t*(15) = 0.195. WT (*n* = 10), cKO (*n* = 7). (**D**) A schematic of fear-based PS test paradigm. Animals were asked to discriminate between similar contexts with or without electric foot shocks. (**E**) Graphs showing the percentage of freezing behavior in training context A and similar context B. Repeated-measures ANOVA was performed. Context A: *P* = 0.969, F(1,7) = 0.002. Context B: *P* = 0.046, F(1,7) = 5.894. WT (*n* = 5), cKO (*n* = 4). (**F**) A graph showing discrimination ratio. LIN28A-cKO mice showed a significant improvement compared with WT mice. Repeated-measures ANOVA was performed. *P* = 0.007, F(1,7) = 14.103. WT (*n* = 5), cKO (*n* = 4). (**G**) A graph showing the freezing percentage when LIN28A WT and LIN28A-cKO mice were exposed to training context A, similar context B, and completely new context C. Student’s paired *t* test was performed. LIN28A WT: context A vs. B, *P*= 0.114, *t*(4) = 1.811; context B vs. C, *P* = 0.013, *t*(4) = 4.244; context A vs. C, *P* = 0.003, *t*(4) = 6.340. LIN28A cKO: context A vs. B, *P*= 0.007, *t*(3) = 6.581; context B vs. C, *P* = 0.992, *t*(3) = 0.010; context A vs. C, *P* = 0.005, *t*(3) = 7.443. WT (*n* = 5), cKO (*n* = 4). Data are presented as mean ± SEM. *P < 0.05, **P < 0.01.

**Figure 5:**
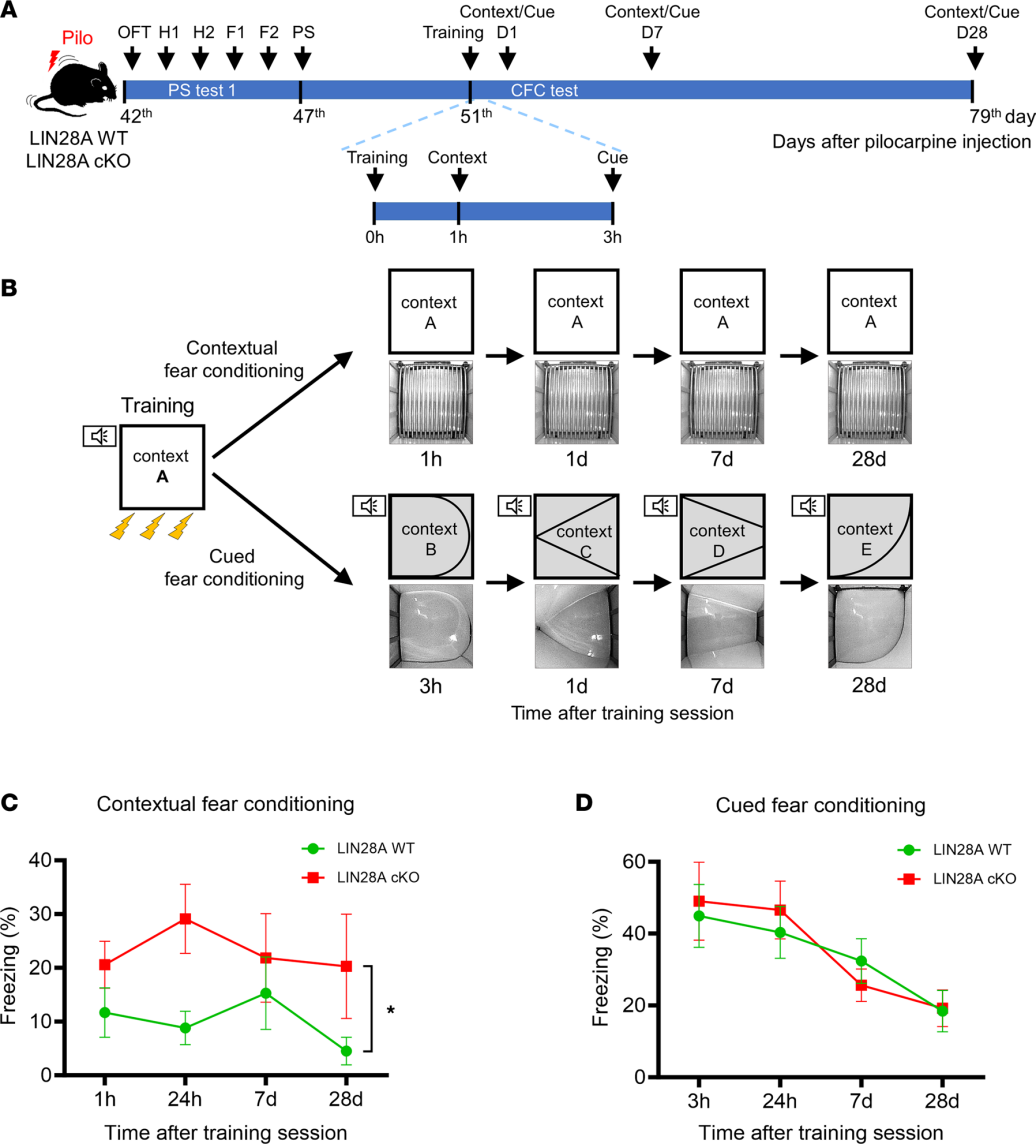
Amelioration of hippocampus-dependent memory impairment in epileptic LIN28A-cKO mice. (**A**) Experimental timeline. (**B**) A schematic illustration of the fear-conditioning paradigm. (**C**) A graph showing the percentage of freezing behavior in contextual fear conditioning test. Epileptic LIN28A-cKO mice showed a higher freezing percentage in response to contextual fear conditioning, indicating attenuation of spatial memory deficits. Repeated-measures ANOVA was performed. *P* = 0.025, F(1,14) = 6.289. WT (*n* = 10), cKO (*n* = 6). (**D**) A graph showing the percentage of freezing behavior in cued fear conditioning test. Epileptic LIN28A WT and LIN28A-cKO mice demonstrated no difference in cued fear conditioning. Repeated-measures ANOVA was performed. *P*= 0.905, F(1,14) = 0.015. WT (*n* = 10), cKO (*n* = 6). Data are presented as mean ± SEM. *P < 0.05.

Because multiple cell types in DG expressed LIN28A after acute seizure, we generated LIN28A-cKO mice in which *Lin28a* was deleted in all neuronal lineages to examine its role in epilepsy. When LIN28A WT and LIN28A-cKO mice were 6 weeks old, pilocarpine was administered to induce acute seizures. Then, at 6 weeks after pilocarpine-induced SE, LIN28A WT and LIN28A-cKO mice were subjected to multiple memory tests including 2 different PS, contextual, and cued fear conditioning tests (Figure 4A and Figure 5A). For the PS test (PS test 1), mice were asked to discriminate between identical objects with subtle differences in orientation placed in different floor patterns (Figure 4B). To do that, animals explored 2 identical rubber dolls positioned in different orientations (both objects showed rear view in familiarization trial 1[F1] and front view in F2) placed on different types of floor patterns (wide grid and narrow grid) in 2 familiarization trials (F1, F2). In the testing phases the next day, each animal was allowed to explore a rear-view object (novel object) and a front-view object (familiar object) placed on the floor pattern used in F2 (Figure 4B) to determine the ability of the mice to distinguish analogous experiences. Based on the discrimination ratio, epileptic LIN28A WT mice showed a poor PS ability, which was significantly ameliorated in LIN28A-cKO mice (Figure 4C). However, the total distance the animals moved was comparable between the 2 groups, indicating no locomotor dysfunction (Figure 4C). We then utilized another PS test (PS test 2) based on fear discrimination between 2 similar contexts (Figure 4D). We first confirmed these 2 contexts were similar enough, as the freezing level between context A and context B on day 1 was comparable in both LIN28A WT and LIN28A-cKO mice (data not shown). When epileptic LIN28A WT and LIN28A-cKO mice were exposed to both context A and context B for 9 consecutive days, both mice showed comparable freezing percentage in context A, but in similar context B, LIN28A WT and LIN28A-cKO mice showed a different freezing behavior (Figure 4E). Further analysis of the discrimination ratio revealed that LIN28A cKO exhibited significantly higher levels of discrimination between the 2 contexts compared with epileptic LIN28A WT mice (Figure 4F). Finally, when the mice were subjected to context C at the end of testing period, epileptic LIN28A WT mice showed a significant reduction in freezing levels compared with contexts A and B (Figure 4G), indicating the preservation of their ability to recognize noticeably different novel environments.

We then performed additional behavioral tests, CFC and cued fear conditioning, to confirm the involvement of LIN28A in hippocampal memory impairment (Figure 5A). After LIN28A WT and LIN28A-cKO mice were exposed to context A with electrical foot shocks and tone pairing (training), spatial contextual memory and cued memory were tested repeatedly by reintroducing the animal to the same context A without a shock or to new contexts B, C, D, and E with an auditory tone (Figure 5B). Compared with epileptic LIN28A WT mice, LIN28A-cKO animals showed a higher freezing percentage from 1 hour to 28 days after training when reintroduced to the same context A (Figure 5C), suggesting that LIN28A cKO ameliorated spatial memory impairment after excitotoxic brain insult. However, cued memory was not different between LIN28A WT and LIN28A-cKO animals (Figure 5D). To examine whether LIN28A deletion itself may influence PS or contextual fear memory, we subjected sham-manipulated LIN28A WT and LIN28A-cKO mice to PS, contextual, and cued fear conditioning tests (Supplemental Figures 1 and 2; supplemental material available online with this article; https://doi.org/10.1172/jci.insight.175627DS1). Interestingly, we found that sham-manipulated animals showed no differences in any of the 4 tests, including 2 different PS, CFC, and cued fear conditioning tests (Supplemental Figure 1 and 2), suggesting a specific role of LIN28A in epilepsy. Taken all together, our data demonstrate that LIN28A deletion in all neuronal lineages can alleviate PS and hippocampal spatial memory impairment after pilocarpine-induced SE but not in sham condition.

### LIN28A deletion in all neuronal lineages reduces the number of hilar EGCs after acute seizure.

**Figure 6:**
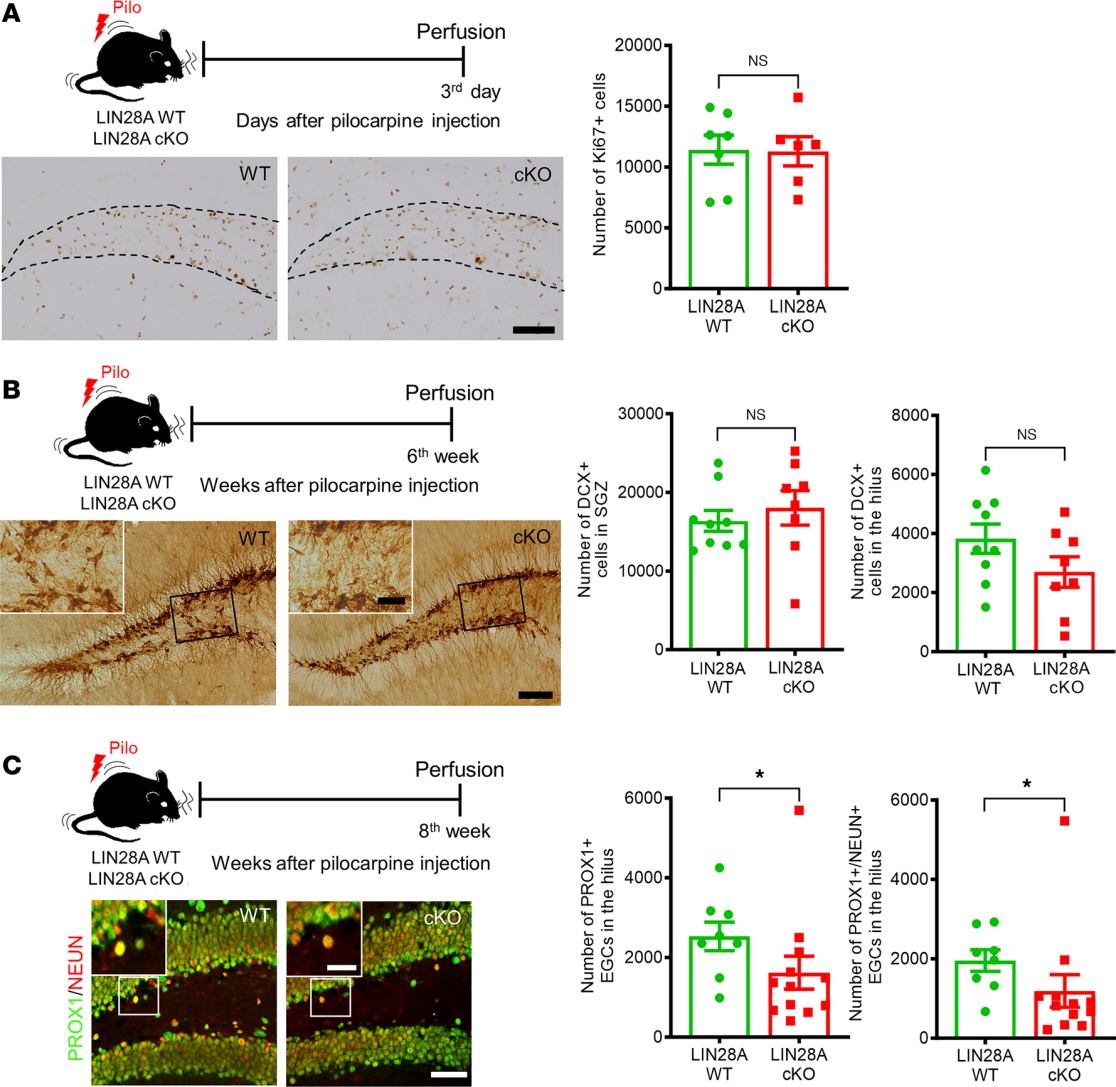
Effects of LIN28A conditional deletion on seizure-induced aberrant hippocampal neurogenesis. (**A**) Three days after pilocarpine-induced status epilepticus (SE), Ki67-expressing cells in the subgranular zone (SGZ) were compared between LIN28A WT and LIN28A-cKO mice. Microscopic images showing Ki67-expressing cells in the SGZ and hilus. The number of Ki67-immunoreactive cells in the SGZ of LIN28A WT and LIN28A-cKO groups was similar. Scale bar: 50 μm. Student’s *t* test, *P* = 0.936, *t*(11) = 0.082. WT (*n* = 7), cKO (*n* = 6). (**B**) Six weeks after acute seizures, DCX expression in the SGZ and the hilus was assessed. The number of DCX^+^ cells in the SGZ and the hilus did not differ significantly between LIN28A WT and LIN28A-cKO mice. Scale bars: 50 μm and 20 μm (low-magnified image and inset, respectively). Student’s *t* test was used. *P* = 0.516, *t*(15) = 0.666 for the left graph, and *P* = 0.136, *t*(15) = 1.577 for the right graph. WT (*n* = 9), cKO (*n* = 8). (**C**) Eight weeks after acute seizures, double immunofluorescence staining for PROX1 and NEUN was used to study hilar ectopic granule cells (EGCs) and their cellular phenotypes in LIN28A WT and LIN28A-cKO mice. LIN28A-cKO mice showed a significant reduction in the number of hilar EGCs compared with WT mice, in addition to the number of PROX1/NEUN-expressing EGCs, suggesting that LIN28A deletion caused a reduction in mature EGCs after acute seizures. Scale bars: 50 μm and 20 μm (low-magnified image and inset, respectively). Mann-Whitney *U* test was used. *P* = 0.003, *U* = 19.500 for the left graph, and *P* = 0.015, *U* = 17.000 for the right graph. WT (*n* = 8), cKO (*n* = 12). Data are presented as mean ± SEM. *P < 0.05.

Since our data demonstrate that epileptic LIN28A-cKO mice exhibited improved PS memory, which is known to be associated with adult hippocampal neurogenesis (26), we then asked whether LIN28A deletion in neuronal lineages could affect aberrant, seizure-induced hippocampal neurogenesis. Three days after SE, the DG contained many Ki67-expressing cells, possibly including proliferating glial cells and neural progenitors (Figure 6A). To measure the proliferative activity of hippocampal progenitors, we counted only the number of Ki67^+^ cells in the SGZ of the DG. Compared with LIN28A WT mice, LIN28A-cKO mice had a similar number of Ki67-immunoreactive cells in the SGZ (Figure 6A), suggesting that LIN28A deletion had no significant effect on proliferative progenitors. Next, we evaluated adult-generated neurons at 6 weeks after pilocarpine using DCX staining. In both LIN28A WT and LIN28A-cKO mice, DCX-immunoreactive cells were detected in the SGZ and hilus in chronic epilepsy (Figure 6B). Moreover, the number of DCX^+^ cells in the SGZ did not differ significantly between the LIN28A WT and LIN28A-cKO groups, although there was a decreasing trend in the number of ectopic DCX^+^ cells in the hilus of the LIN28A-cKO group (Figure 6B). However, when we assessed PROX1-expressing EGCs in the hilus of DG at 8 weeks after pilocarpine injection, we found that the number of ectopic granule neurons in the LIN28A-cKO group had decreased significantly (Figure 6C). Because LIN28A deletion in neuronal lineages attenuated the seizure-induced generation of hilar EGCs without affecting the production of DCX-expressing neuroblasts and immature granule neurons, we further evaluated PROX1/NEUN-expressing mature granule cells in the hilus and found that their numbers were significantly decreased in LIN28A-cKO mice (Figure 6C). Together, our findings indicate that LIN28A deletion in the neuronal lineage can alleviate seizure-induced aberrant hippocampal neurogenesis, especially the formation of mature ectopic granule neurons in the hilus.

### Transcriptomics analysis demonstrates that serotonin receptors, including Htr4, are altered by LIN28A cKO after pilocarpine-induced SE.

**Figure 7:**
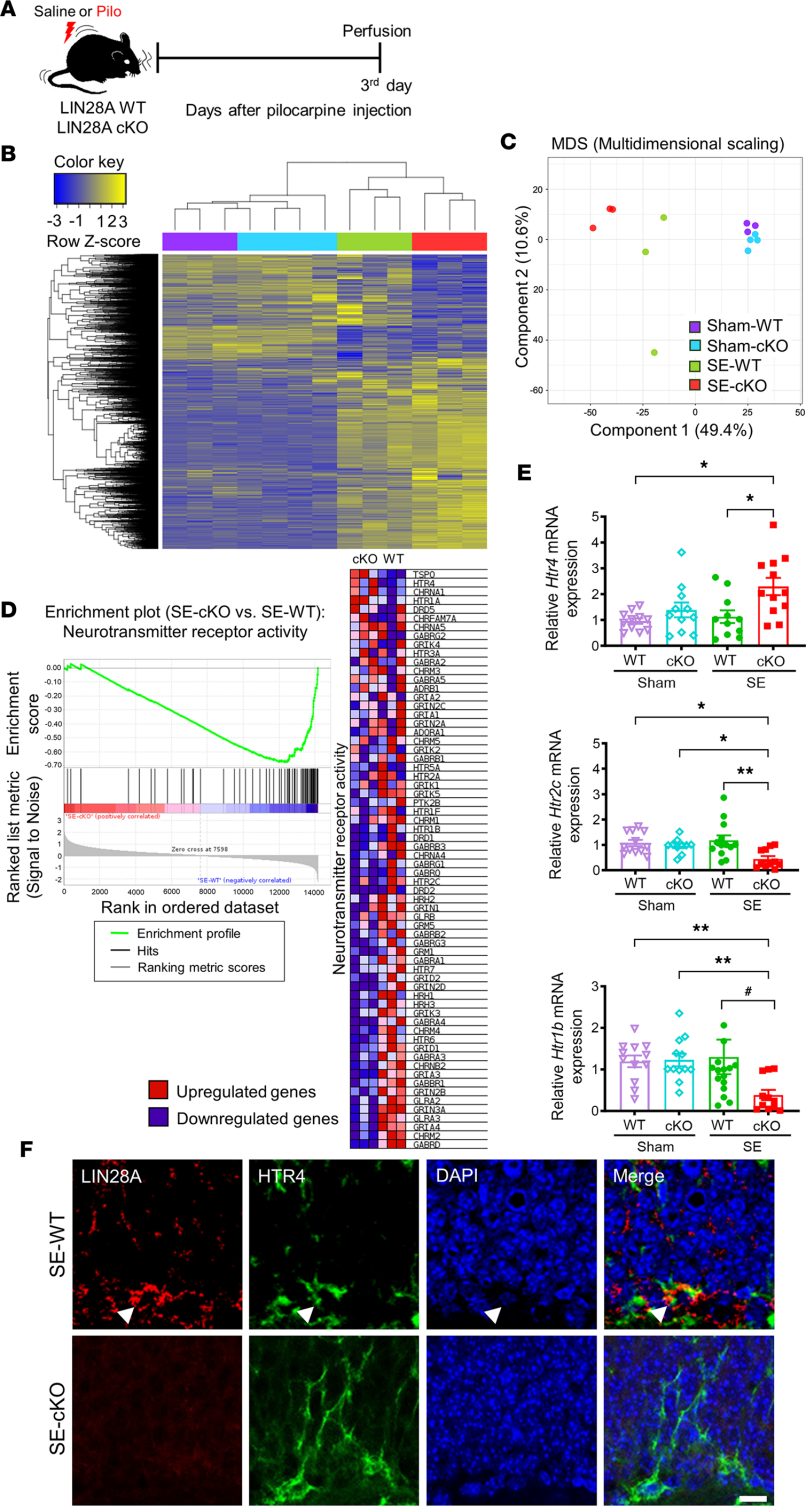
Transcriptomics analysis of hippocampi from LIN28A cKO WT and -cKO mice at 3 d after pilocarpine-induced status epilepticus (SE). (**A**) Experimental timeline. (**B**) Hierarchical clustering between LIN28A WT and LIN28A-cKO mice. (**C**) Multidimensional scaling plot demonstrating 3 distinct groups among sham-WT, sham-cKO, SE-WT, and SE-cKO. (**D**) GSEA enrichment plot and heatmap of differentially expressed genes (DEGs) with neurotransmitter receptor activity in pilocarpine-treated LIN28A WT and LIN28A-cKO mice. (**E**) Graphs showing the relative mRNA expression of several DEGs in the hippocampus. Note that *Htr4* expression was significantly increased, whereas *Htr2c* and *Htr1b* transcription was downregulated in pilocarpine-treated LIN28A-cKO compared with WT mice. *Htr4*: Welch’s ANOVA test without outlier, followed by 2-stage linear step-up procedure of Benjamini, Krieger, and Yekutieli post hoc test. *P* = 0.010, W(3.000, 20.500) = 4.835. Sham-WT (*n* = 12), sham-cKO (*n* = 11), SE-WT (*n* = 11), SE-cKO (*n* = 12). *Htr2c*: Kruskal-Wallis *H* test without outlier, followed by 2-stage linear step-up procedure of Benjamini, Krieger, and Yekutieli post hoc test. *P* = 0.005, *H* = 12.480. Sham-WT (*n* = 12), sham-cKO (*n* = 11), SE-WT (*n* = 13), SE-cKO (*n* = 12). *Htr1b*: Kruskal-Wallis *H* test without outlier, followed by 2-stage linear step-up procedure of Benjamini, Krieger, and Yekutieli post hoc test. *P* = 0.001, *H* = 16.060. For pilocarpine-treated animals, additional Mann-Whitney *U* test was performed. *P* = 0.014, *U* = 47.500. Sham-WT (*n* = 12), sham-cKO (*n* = 13), SE-WT (*n* = 17), SE-cKO (*n* = 12). Data are presented as mean ± SEM. *P < 0.05, **P < 0.01,^#^*P* < 0.05 vs. SE-WT. (**F**) Representative microscopic images showing HTR4- and LIN28A-immunoreactive cells in the subgranular zone (SGZ) of pilocarpine-treated LIN28A WT and LIN28A-cKO mice. White arrowheads indicate a double-immunoreactive cell in SGZ. Experiment was independently replicated 3 times. Scale bar: 20 μm.

Since seizure-induced LIN28A upregulation was observed in the early stages after acute seizure, RNA-Seq was performed using hippocampi from LIN28A WT and LIN28A-cKO mice at 3 days after saline or pilocarpine injection to identify differentially expressed genes (DEGs) (Figure 7A). Hierarchical clustering demonstrated a clear difference in gene expression patterns between sham and SE, in addition to LIN28A WT and LIN28A-cKO groups (Figure 7B). Moreover, the multidimensional scaling (MDS) plot demonstrated that, compared with sham-manipulated LIN28A WT and LIN28A-cKO groups showing a relative similarity, pilocarpine-treated LIN28A WT and LIN28A-cKO displayed distinct transcriptomics differences (Figure 7C), in line with our hierarchical clustering. When we further analyzed the DEGs between SE-treated LIN28A WT and LIN28A-cKO groups using the gene set enrichment analysis (GSEA) tool, we found that genes associated with neurotransmitter receptor activity were significantly altered by LIN28A deletion after SE (Figure 7D). Furthermore, qPCR validation revealed that, after SE, the expression of hippocampal *Htr4* was significantly increased, whereas *Htr2c* and *Htr1b* expressions were reduced in LIN28A-cKO mice (Figure 7E). Finally, we confirmed that HTR4 immunoreactivity was observed in LIN28A-expressing cells in SGZ at 3 days after SE (Figure 7F), suggesting that HTR4 can be a potential LIN28A-downstream target in epilepsy. Collectively, our results demonstrate that LIN28A deletion in all neuronal lineages can alter serotonin-mediated signaling after SE, and this can alleviate seizure-induced aberrant neurogenesis and PS memory deficit in epilepsy.

## Discussion

Identification of the critical molecules that modulate aberrant hippocampal neurogenesis is crucial for understanding complex hippocampal cognitive dysfunction under pathological conditions, since physiologic adult-generated granule neurons can contribute to distinguishing subtle differences between similar experiences — referred to as PS (2). In the present study, we show that blocking LIN28A induction after pilocarpine-induced SE reduced the number of hilar EGCs and attenuated hippocampal memory impairment. For spatial PS evaluation, we devised a potentially novel spatial PS test based on modification of an episodic memory–based PS test (27, 28). When animals were asked to discriminate a novel object based on the combination of a floor pattern and 2 identical objects in different orientations (front view or rear view), LIN28A WT mice had difficulty identifying the novel object, implying impaired PS ability. However, reduced seizure-induced EGC production by LIN28A deletion led to a significant improvement in PS capability. We also demonstrated a PS improvement in LIN28A cKO when another classical PS test based on fear discrimination between 2 similar contexts was utilized (26), demonstrating a critical role for abnormal newborn neurons in PS memory dysfunction. This hypothesis was supported by a DG-cornu Ammonis 3 (DG-CA3) computational model demonstrating that addition of a small population of hilar EGCs was sufficient to disrupt simulation of PS (29). Interestingly, sham-manipulated WT and cKO animals showed similar intact PS capability, suggesting a specific role of LIN28A in epilepsy. To further confirm the involvement of LIN28A in general hippocampal memory in epilepsy, we performed contextual and cued fear conditioning tests and demonstrated improvement in spatial memory tasks in LIN28A-cKO mice. Consistent with our findings, pharmacological approaches to suppress seizure-induced neurogenesis by valproic acid or endoneuraminidase administration spared hippocampal spatial memory deficits, accompanied by the restoration of features of aberrant hippocampal neurogenesis (11, 12). Moreover, a genetic approach to selectively ablate adult neurogenesis before seizure activity normalized hippocampus-dependent spatial memory impairment (10), corroborating our findings. It’s noteworthy to mention that LIN28A depletion in other brain regions may influence epilepsy-associated cognitive changes, given the use of the *Nestin-Cre* system in our study. Finally, LIN28A did not influence memory extinction based on assessment of memory retrieval for 28 days after pilocarpine-induced SE. A previous study reported no effect of adult-generated granule neurons on extinction of contextual fear memory (30). Taken together, our data demonstrate that LIN28A is a potentially novel regulator of seizure-induced aberrant hippocampal neurogenesis and affects newborn neuron-associated memory function in epilepsy.

Because LIN28A induction after acute seizures was observed in subgranular cells, we comprehensively analyzed the effects of LIN28A modulation on seizure-induced abnormal hippocampal neurogenesis. Even though the number of Ki67^+^ proliferating progenitors did not differ between the LIN28A WT and LIN28A-cKO groups, we found that LIN28A deletion in the neuronal lineage could reduce the production of hilar EGCs in chronic epilepsy. Moreover, when we evaluated mature EGCs using double immunofluorescence, LIN28A deletion downregulated the number of PROX1/NEUN-expressing mature granule neurons after seizure activity. Since 2 major roles of LIN28A are the promotion of cellular growth and survival (31, 32), our data indicate that LIN28A can play a critical role in the survival of newborn neurons, especially the generation of mature EGCs in epilepsy. It is a bit surprising that the proliferative activity of neural progenitors was unaffected by LIN28A deletion, in contrast to previous reports showing that LIN28A had a pro-proliferative function under physiological conditions (15, 18, 33). We offer 2 possible reasons: (a) seizure-mediated enhanced mitosis outweighs the inhibition of proliferation by LIN28A deficiency, or (b) LIN28A does not affect the proliferation of progenitors in the epileptic context, unlike in physiological conditions. More elaborate studies will be required to conclude the effects of LIN28A on hippocampal proliferation after acute seizures. Collectively, our data show that blocking LIN28A induction after acute seizure attenuated seizure-induced aberrant hippocampal neurogenesis, especially the generation of mature EGCs.

LIN28A is thought to inhibit *let-7* miRNA biogenesis and thereby negatively regulate the translation of *let-7* target genes (31, 32). Because *Lin28a* mRNA is, itself, a *let-7* target molecule (34), changes in *let-7* expression can alter the level of LIN28A protein. Intriguingly, a couple of studies have reported that multiple *let-7* isoforms were decreased in the hippocampus at specific periods following SE (35, 36). These data raise the possibility that the downregulation of *let-7* miRNA after acute seizures could unleash the translation of LIN28A, resulting in the induction of LIN28A protein in the SGZ of the DG.

To investigate the molecular mechanisms associated with the role of LIN28A after acute seizure, we performed transcriptomics analysis using hippocampi from LIN28A WT and LIN28A-cKO mice at 3 days after SE. Among many potential target genes identified by RNA-Seq, we found that the expression of *Htr1b* and *Htr2c* was significantly reduced, whereas that of *Htr4* was increased in LIN28A-cKO mice. Regarding the effects of serotonin receptors on hippocampal neurogenesis, 1 study reported decreased survival of adult-generated neurons in HTR1A and HTR1B double mutants (37). HTR1B is associated with learning and memory; an HTR1B agonist effectively reduced cognitive function in rats (38). In relation to memory, administration of the HTR2C antagonist SB242084 enhanced reversal learning (39), while administration of the HTR2C antagonist RS-102221 attenuated stressor-induced spatial memory deficits (40), suggesting that blocking HTR2C benefits hippocampal memory function. These findings support those of the current study: decreased *Htr1b* and *Htr2c* expression in LIN28A-cKO mice was associated with improved performance in spatial memory tasks. HTR4 also is thought to regulate adult hippocampal neurogenesis (41), but the molecular mechanisms by which it regulates neurogenesis might be more complicated than expected, since HTR4 KO blocked fluoxetine-mediated neurogenic effects without affecting baseline neurogenesis (42). Moreover, *Htr4* is predicted to be a *let-7a* miRNA target (miRDB, microRNA target prediction database; http://mirdb.org/), and an inverse relationships between *let-7a* and *Htr4* was reported in anhedonia (43), implying that HTR4 is involved in LIN28A/*let-7* signaling. Additionally, valproic acid under physiologic conditions can inhibit the serotonin system by activating monoamine oxidase A (44) and can promote neuronal survival through epigenetic mechanisms (45). Since valproic acid is known to affect cognitive function (46), it would be intriguing to investigate whether cognitive improvement observed in LIN28A cKO might be reversed by the administration of valproic acid following acute seizures. Taken altogether, we identified downstream target molecules induced by LIN28A deletion after seizure activity — i.e., *Htr1b*, *Htr2c*, and *Htr4* — which are all involved in adult hippocampal neurogenesis or cognitive function. Future studies to assess the precise mechanisms of LIN28A signaling are required.

In summary, we determined the spatiotemporal expression pattern of *Lin28a* transcript and protein after pilocarpine-induced SE. We also identified cellular phenotypes of seizure-induced LIN28A–expressing cells in the adult SGZ of the DG. When we blocked LIN28A induction in neuronal lineages using a genetic approach, we found that LIN28A deletion alleviated hippocampal memory impairment in chronic epilepsy by enhancing spatial PS. Additionally, we found a reduction in mature hilar EGCs in LIN28A-cKO mice, suggesting a role of LIN28A in seizure-induced aberrant hippocampal neurogenesis. To identify LIN28A-mediated molecular mechanisms, we performed transcriptomics analysis, resulting in identification of the potential LIN28A downstream targets of *Htr1b*, *Htr2c*, and *Htr4* and further IHC validation of HTR4 in LIN28A-expressing cells after acute seizures. Collectively, we identified LIN28A as a modulator of abnormal, seizure-induced hippocampal neurogenesis, affecting newborn granule neuron–related spatial memory function. The results of this study provide essential basic information for understanding hippocampal memory dysfunction in epilepsy and other brain diseases involving the hippocampus, such as schizophrenia, traumatic brain injury, and Alzheimer’s disease.

## Methods

### Animals.

Male C57BL/6N mice (6 weeks old, Koatech) were used to assess spatiotemporal LIN28A expression patterns. For the functional analysis of LIN28A in epilepsy, we crossed *Nestin-Cre* mice (47) with *Lin28a^loxP/+^* mice (a gift from Hao Zhu at UT Southwestern Medical Center, Dallas, Texas, USA), followed by the breeding of *Nestin-Cre:Lin28a^loxP/+^* mice with *Lin28a^loxP/+^* mice to generate male and female LIN28A-cKO and LIN28A WT mice. Mice were genotyped by polymerase chain reaction (PCR) using genomic DNA and primers for *Lin28a* (forward: 5′-TCC AAC CAG CAG TTT GCA G-3′, reverse: 5′-GCA GCT GGT AAG AAC AAA CCT-3′), and *Nestin-Cre* (forward: 5′-GGT CGA TGC AAC GAG TGA TGA GG-3′, reverse: 5′-GCT AAG TGC CTT CTC TAC ACC TGC G-3′). LIN28A WT and LIN28A-cKO mice were backcrossed to C57BL/6N mice for at least 5 generations prior to beginning the studies. All mice were bred and housed in an animal facility with a 12-hour light, 12-hour dark cycle and had access to food and water ad libitum.

### Pilocarpine-induced mouse model of temporal lobe epilepsy.

Pilocarpine-induced SE was established as previously described (48–50). Briefly, C57BL/6N, LIN28A WT, and LIN28A-cKO mice at 6 weeks of age were given scopolamine methyl nitrate (i.p.; 2 mg/kg; Sigma-Aldrich, S2250) and terbutaline hemisulfate salt (i.p.; 2 mg/kg; Sigma-Aldrich, T2528) to block the peripheral effects of pilocarpine and dilate the respiratory tract, respectively. Thirty minutes later, pilocarpine hydrochloride (i.p.; Sigma-Aldrich, P6503) was injected at 260 mg/kg for male LIN28A transgenic mice and 280 mg/kg for both female LIN28A transgenic and male C57BL/6N mice; then, mice were placed in an incubator that was maintained at 31˚C (ThermoCare). Acute seizures were behaviorally monitored using a modified Racine’s scale (51) (stage 1: mouth and facial movement; stage 2: head nodding; stage 3: forelimb clonus; stage 4: rearing with forelimb clonus; stage 5: rearing and falling with forelimb clonus). Once SE was initiated (defined by continuous generalized convulsive seizures), mice were placed at room temperature for 3 hours and returned to the incubator after behavioral seizure activity was quenched with diazepam (10 mg/kg; Sigma-Aldrich, D0899). Only mice showing SE were selected for further processing. Mice were given 5% dextrose solution (i.p.; 1 mL) to facilitate their recovery. At 2 days after pilocarpine injection, the mice were returned to their cages and randomly assigned to the experiments.

### qPCR.

Mice were anesthetized and briefly perfused transcardially with cold saline. Total RNA from the hippocampi was isolated using TRIzol reagent according to the manufacturer’s instructions. In brief, tissues were frozen in liquid nitrogen, pulverized, homogenized in 1 mL of TRIzol reagent, and incubated for 5 minutes at room temperature. Then, 200 mL of chloroform was added, vigorously mixed, and incubated for 3 minutes at room temperature. Samples were centrifuged (19,320*g* for 20 minutes), and the aqueous phases were transferred to fresh tubes with equal volumes of isopropanol and incubated on ice for 30 minutes. After centrifugation at 19,320*g* for 15 minutes, the RNA pellets were washed in 75% ethanol and dissolved in RNase-free water. RNA qualities and concentrations were assessed by Nanodrop (Thermo Fisher Scientific). Then, cDNA was generated using a ReverTra Ace qPCR RT Master Mix with gDNA Remover (Takara), followed by qPCR with SYBR green (Takara) and an MX3005P system (Stratagene). The primer sequences used were forward: 5′-CAA CGG GTT GTG ATG ACA GGC AAA-3′, reverse: 5′-TAG TGC AGT TGG CAT CCT GGA GAA-3′for *Lin28a*, forward: 5′-CGC CGA CGG CTA CAT TTA C-3′, reverse: 5′-TAG CTT CCG GGT CCG ATA CA-3′ for *Htr1b*, forward: 5′-CTA ATT GGC CTA TTG GTT TGG CA-3′, reverse: 5′-CGG GAA TTG AAA CAA GCG TCC-3′ for *Htr2c*, forward: 5′-AGT TCC AAC GAG GGT TTC AGG-3′, reverse: 5′-CAG CAG GTT GCC CAA GAT G-3′ for *Htr4*, and forward: 5′-TCA ACA GCA ACT CCC ACT CTT CCA-3′, reverse: 5′-ACC CTG TTG CTG TAG CCG TAT TCA-3′for *Gapdh*. Quantitative analysis of mRNA expression using the ΔΔCt method was carried out as previously described (52, 53).

### Histologic assessments.

Mice were anesthetized and perfused transcardially with cold 4% paraformaldehyde (PFA) in 0.1M PBS. The brains were removed and postfixed in 4% PFA overnight, before being cryoprotected in 30% sucrose in 0.1M PBS. The brains were bisected, and the half-brains were coronally sectioned 30 μm thick using a cryostat. In situ hybridization was performed using riboprobes for *Lin28a* (NM_145833.1, nucleotides 200–674). Sense and antisense riboprobes for *Lin28a* were labeled with DIG by in vitro transcription using a DIG RNA labeling kit (Roche). Tissue sections were hybridized with sense or antisense probes diluted in hybridization solution (150 ng/mL) at 53˚C for 18 hours and then incubated with a biotin-conjugated mouse anti-DIG antibody (1:500, Jackson ImmunoResearch, 200-062-156), followed by cy3-conjugated streptavidin (1:500, Jackson ImmunoResearch, 016-160-084). Then, immunofluorescence of the sections was induced with antibodies to GFAP (1:1000, MilliporeSigma, MAB360) and IBA1 (1:500, Wako Pure Chemical Industries, 019-19749) at 4˚C overnight. The next day, the sections were incubated with cy5–conjugated anti-mouse IgG (Jackson ImmunoResearch, 715-175-150) and cy2-conjugated anti–rabbit IgG (Jackson ImmunoResearch, 711-225-152). The triple-labeled sections were visualized with a confocal microscope (LSM900; Carl Zeiss Microscopy) and imaged with pseudo-coloring. For IHC, tissue sections were either mounted on charged slides or free floated in 0.01M PBS. Slides underwent antigen retrieval using 0.01M citric acid, pH 6.0, at 100˚C for 15 minutes, followed by 12 minutes in tris-buffered saline (TBS) at room temperature. The free-floating tissue sections underwent the same staining procedure, except that the antigen retrieval step was omitted. For visualization with 0.1% diaminobenzidine tetrahydrochloride (DAB), we removed endogenous peroxidase activity by incubating sections with 0.3% H_2_O_2_ for 30 minutes at room temperature. Nonspecific binding was blocked with 3% normal donkey serum (Jackson ImmunoResearch, 017-000-121) and 0.3% Triton X-100 in TBS for 1 hour at room temperature. The primary antibodies in this study were chosen based on the validation results provided by the manufacturer: rabbit anti-LIN28 (1:200, Santa Cruz Biotechnology, sc-67266), mouse anti-GFAP (1:1,000, MilliporeSigma, MAB360), mouse anti-nestin (1:50, MilliporeSigma, MAB353), guinea pig anti-doublecortin (DCX, 1:2,000, MilliporeSigma, AB2253), mouse anti-calretinin (1:100, MilliporeSigma, MAB1568), mouse anti-NEUN (1:200, MilliporeSigma, MAB377), rabbit anti-Ki67 (1:500, Thermo Fisher Scientific, MA5-14520), rabbit anti-PROX1 (1:500, MilliporeSigma, ABN278), and mouse anti-HTR4 (1:200, Santa Cruz Biotechnology, sc-376158). For double labeling, primary antibodies were simultaneously incubated (LIN28A/GFAP, LIN28A/Nestin, LIN28A/DCX, LIN28A/Calretinin, LIN28A/NEUN, PROX1/NEUN, and LIN28A/HTR4), and cy3- and Alexa Fluor 488–conjugated fluorescent secondary antibody incubations were further processed for each antibody (1:500, Jackson ImmunoResearch, 711-165-152, 711-545-152, 715-545-151, 715-165-150, 706-546-148). Finally, DAPI (50 μg/mL, Roche, 10 236 276 001) was counterstained. For LIN28A, Ki67, and DCX staining, after the labeling with secondary antibodies (Jackson ImmunoResearch, 111-036-003, 106-036-003), the sections were visualized with 0.1% DAB and examined under an upright microscope (BX51; Olympus). For fluorescence images, sections were mounted with ProLong Gold antifade mountant (Thermo Fisher Scientific, P10144) and assessed under a confocal microscope (LSM900; Carl Zeiss Microscopy).

### Microscopic analysis and quantification.

Quantitative analysis was performed by an observer blinded to the experimental group. In situ hybridization to *Lin28a* and IHC to GFAP or IBA1 was analyzed using the color threshold function of PyCharm software (a gift from Hyun-Jong Jang at the Catholic University of Korea). Briefly, after the DG pixel area was measured, the area of both red channel pixels greater than 80 pixels and green channel pixels greater than 90 pixels was determined as the yellow double-positive signal areas. Data are reported as the percentage of double-fluorescence signal areas in DG. For the percentage of LIN28A immunoreactivity in DG, NIH ImageJ software was utilized. The pixel intensity of the molecular layer in the tissue section was measured as the background intensity. Using the threshold function, the area of pixels greater than the background intensity was measured as the LIN28A staining area. Then, similar to the analysis of in situ hybridization, after DG pixel area was measured, the percentage of LIN28A immunoreactive areas in DG was presented. For LIN28A-expressing cellular phenotypic analysis, confocal images in the Z planes were scanned to quantify marker-positive cells, and the percentage of double-stained cells divided by the number of LIN28A-immunoreactive cells was presented. Finally, for the analysis of aberrant hippocampal neurogenesis, immunoreactive cells were quantified as previously described (54). The SGZ and hilar zone were defined as the area within and beyond the diameter of 1 granule cell from the margin of the GCL, respectively. All the immunoreactive cells were counted in every 12th coronal section throughout DG, and the numbers counted in each section were added before being multiplied by 24 to estimate the total number of cells in each animal.

### RNA-Seq.

At 3 days after pilocarpine-induced SE, hippocampi from LIN28A WT and LIN28A-cKO mice were isolated. After RNA extraction, libraries were generated using TruSeq stranded total RNA LT sample prep kits (Illumina). Using an Illumina platform, preprocessed raw reads were aligned to the *Mus musculus* genome (mm10) using HISAT v2.1.0. After alignment, the relative abundance of genes was measured in Read Count using StringTie v2.1.3b. We determined DEGs using estimates of gene abundance in each sample (GEO accession no. GSE246519). For the DEG set, hierarchical clustering and MDS analysis were performed using complete linkage and Euclidean distance as a measure of similarity. Gene enrichment and functional annotation analysis in addition to pathway analysis for significant gene lists were performed based on KEGG pathways and GSEA.

### Memory tests.

PS and fear conditioning tests were conducted 6–10 weeks after saline or pilocarpine injection. Two different PS tests were performed as described previously with minor modifications (26, 28). Briefly, animals underwent 3 rounds of habituation, 2 rounds of familiarization, and a test trial each day. All trials were performed in a white, square, open-field box (44 × 44 × 30 cm) under dim light (60 lux) between 7 a.m. and 9 a.m. On the first day of habituation, locomotor activity was assessed by calculating the distance moved in the open-field arena for 15 minutes using a video tracking system (SMART 3.0, Panlab). After 2 additional rounds of habituation trials, animals were allowed to explore 2 identical objects (rubber doll, rear view) on a wide grid floor pattern (F1). The next day, animals were exposed to the same objects from a different view (front) on a narrow grid floor pattern (F2). To help mice to distinguish this difference, a star-shaped picture was attached in the middle of the wall near the objects, at a height of 15 cm. Familiarization sessions ended when the mice explored both objects for a total of 30 seconds or reached 20 minutes for the session. On the PS testing day, mice were placed in the open-field arena, where 1 object with the rear view (novel object) and the other object with the front view (familiar object) were located on a narrow grid floor, and the mice were allowed free exploration for 10 minutes. Then, the discrimination ratio was calculated by subtracting the time spent exploring the familiar object from the time spent exploring the novel object and dividing this value by the total time spent exploring the 2 objects. For the additional PS paradigm, animals were asked to distinguish between 2 similar contexts: context A with foot shock and context B without shock. Basically, mice were placed in a chamber (18 × 17.5 × 32 cm) equipped with a speaker, a video camera, and grids through which electric foot shock (0.5 mA) was delivered (Coulbourn). Context A and similar context B shared many features, including 3 walls of the chamber and the roof. The similar context B differed from context A in 4 aspects. First, a white-coated paper covered the stainless-steel grid floor, along with a white-coated paper featuring diagonal pattern drawings to cover the back wall. Second, the fan and lights were off. Third, the chamber door was left slightly open. Finally, a mild mint scent was added as an olfactory cue. For discrimination learning, mice were exposed to the training context A on day 0, where a single foot shock lasting 2 seconds was delivered at the end of a 6-minute session. Twenty-four hours later, mice were reintroduced to context A with shock, followed by placement in the similar context B without shock after 3 hours of resting in their home cage. From day 2 to day 9, mice were exposed to both context A and B with random order, and freezing behavior for the initial 180 seconds was scored automatically by video-based motion detection software (bout length, 1 second; FreezeFrame, Med Associates Inc.), and the percentage of time freezing was calculated. Fear-based discrimination ratio was calculated by the following equation: (Freezing_Training context_ % – Freezing_Similar context_ %)/(Freezing_Training context_ % + Freezing_Similar context_ %). On day 10, mice were placed in a completely new context C with circular walls and a white-coated paper floor for 6 minutes to see if epileptic WT mice could recognize a noticeably different context, despite having failed to discriminate between 2 similar contexts. For the fear conditioning test, animals were placed in the same chamber (18 × 17.5 × 32 cm) with a speaker, a video camera, and grids for giving electric foot shock (Coulbourn). On the training day, mice were permitted 180 seconds to explore context A before receiving the stimulus. Then, animals received 3 presentations of auditory tones (1,000 Hz, 80 dB, 18 seconds), followed by electrical foot shocks (0.5 mA) for 2 seconds at the end of the tone, with a 1-minute interval between shocks. For CFC, mice were reintroduced to the same context A for 180 seconds 1 hour after training. Two hours later, mice were exposed to the new context B for 6 minutes and were exposed to the same auditory tones for the last 3 minutes. The evaluation of freezing behavior and percentage of time freezing was performed in the same manner as the fear-based PS test. These experiments were repeated at 1 day, 7 days, and 28 days after training to assess memory retrieval.

### Statistics.

All data except for fear-based experiments were expressed as bar graphs with individual data points. All data were presented as mean ± SEM. Experimental groups were assigned by simple randomization. No statistical methods were used to predetermine sample sizes, but our sample sizes are similar to those reported in previous publications (55, 56), and the exact sample size for each group is indicated in the figure legends. To reduce the experimental bias and enhance the reproducibility, behavioral tests were performed by individuals blinded to the genotypes. Data that passed our selection criteria were included for statistical analysis. SPSS statistics version 21 (IBM) or GraphPad Prism 10 software (GraphPad Software Inc.) was used for repeated-measures 1-way ANOVA and the remaining statistical comparisons, respectively. Each statistical analysis method is presented in the figure legends. Basically, a data set with a normal distribution was evaluated by 2-tailed Student’s *t* test or 1-way ANOVA, with a 2-stage linear step-up procedure of Benjamini, Krieger, and Yekutieli post hoc test. If equal variances were not assumed, Welch’s 1-way ANOVA test was performed. In case a normal distribution was not assumed, Mann-Whitney *U* test or Kruskal-Wallis *H* test with 2-stage linear step-up procedure of Benjamini, Krieger, and Yekutieli post hoc test were performed. For fear-based PS and contextual/cued conditioning, repeated-measures ANOVA was utilized. To assess the discrimination capability among context A, B, and C in fear-based PS test, a Student’s paired *t* test was performed. *P* < 0.05 was considered significant.

### Study approval.

Animal experiments were performed in compliance with the animal care guidelines issued by the NIH and by the IACUC at the Catholic University of Korea (approval no. CUMS-2017-0116-03).

### Data availability.

Values for all data shown in the graphs can be found in the Supporting Data Values file. RNA-Seq data are available in GEO database (accession no. GSE246519). Other materials and reagents are available upon request to the corresponding author.

## Author contributions

SYK, JH, and KOC conceptualized and designed the study. IYC and KOC performed the experiments and wrote the manuscript. JHC provided study material. KOC analyzed and interpreted the data.

## Supplementary Material

Supplemental data

Supporting data values

## Version 1 01/09/2024

Electronic publication
