# Supplementary material for: Seizure-induced LIN28A disrupts pattern separation via aberrant hippocampal neurogenesis
Source: JCI Insight. 2024 Jan 9;9(1):e175627. doi: 10.1172/jci.insight.175627 (PMC10906464; doi:10.1172/jci.insight.175627)
Supplement: Supplemental data [file jciinsight-9-175627-s167.pdf]

Supplemental information

Seizure-induced LIN28A disrupts pattern separation via aberrant hippocampal neurogenesis

In-Young Choi,[1] Jung-Ho Cha,[2] Seong Yun Kim,[1,3,4] Jenny Hsieh,[5,6] and Kyung-Ok Cho[*,1,3,4,7,8]

[1]Department of Pharmacology, College of Medicine, The Catholic University of Korea, Seoul 06591, Republic of Korea

[2]Department of Anatomy, College of Medicine, The Catholic University of Korea, Seoul 06591, Republic of Korea

[3]Department of Biomedicine & Health Sciences, The Catholic University of Korea, Seoul 06591, Republic of Korea

[4]Catholic Neuroscience Institute, The Catholic University of Korea, Seoul 06591, Republic of Korea

[5]Department of Neuroscience, Developmental and Regenerative Biology, The University of Texas at San Antonio, San Antonio, TX 78249, USA

[6]Brain Health Consortium, The University of Texas at San Antonio, San Antonio, TX 78249, USA

[7]Institute for Aging and Metabolic Diseases, The Catholic University of Korea, Seoul 06591, Republic of Korea

[8]CMC Institute for Basic Medical Science, the Catholic Medical Center of The Catholic University of Korea, Seoul, 06591, Republic of Korea

*Correspondence to Dr. Kyung-Ok Cho, MD, PhD

Department of Pharmacology, College of Medicine,

The Catholic University of Korea

222 Banpo-daero, Seocho-gu, Seoul 06591, Republic of Korea

Tel: +82-2-3147-8355, Fax: +82-2-536-2485

E-mail: kocho@catholic.ac.kr

Supplemental figure 1

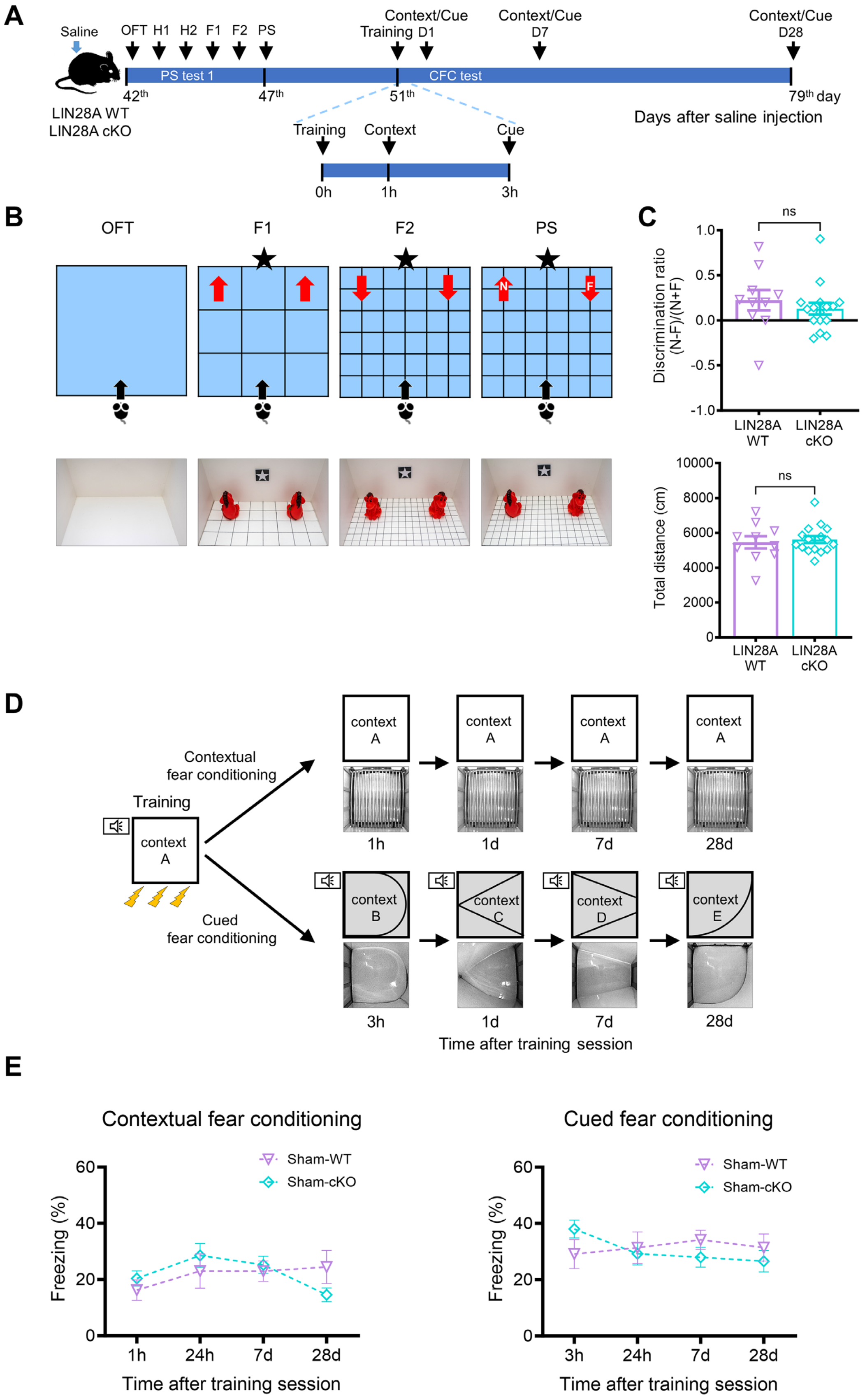

Supplemental Figure 1. No difference in newborn neuron- and hippocampus-associated memory function between sham-manipulated LIN28A WT and cKO mice. (A) Experimental timeline. Animals with chronic epilepsy were subjected to an open-field test (OFT), pattern separation (PS) test 1, and fear conditioning test. **(B)** A schematic presentation of OFT and PS test 1. **(C)** Graphs showing the discrimination ratio and the distance moved. The LIN28A WT and cKO mice showed no difference in their ability to recognize a novel object from analogous experiences. Discrimination ratio: Mann-Whitney U test was performed. $P = 0.151$, U = 52.500. WT (n = 10), cKO (n = 16); distance moved: Student's *t*-test was performed. $P = 0.680$, $t(24) = 0.417$. WT (n = 10), cKO (n = 16). **(D)** A schematic illustration of the fear conditioning paradigm. **(E)** Graphs showing the percentage of freezing behavior in contextual and cued fear conditioning tests. The LIN28A WT and cKO mice showed a similar freezing percentage in response to contextual and cued fear conditioning, indicating intact hippocampal memory function. Repeated measures ANOVA was performed. Contextual fear conditioning: $P = 0.199$, $F(1,19) = 0.011$. WT (n = 9), cKO (n = 12); cued fear conditioning: $P = 0.829$, $F(1,19) = 0.048$. WT (n = 9), cKO (n = 12). Data are presented as mean ± SEM. ns, not significant.

Supplemental figure 2

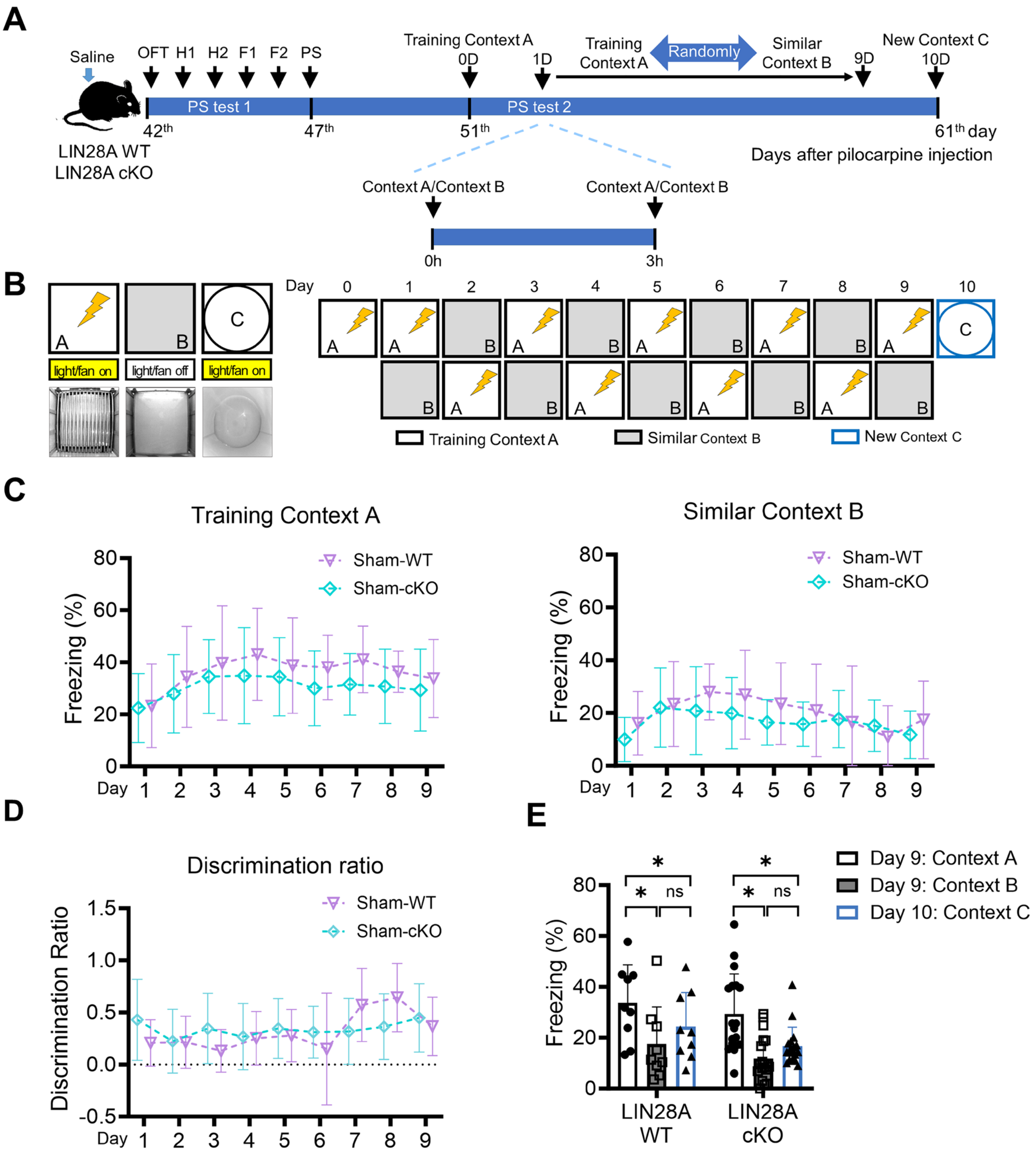

Supplemental Figure 2. No difference in fear-based pattern separation between sham-manipulated LIN28A WT and cKO mice. (A) Experimental timeline. Saline-injected sham animals were subjected to an open-field test (OFT), pattern separation (PS) test 1, and PS test 2. **(B)** A schematic presentation of PS test 2. **(C)** Graphs showing the percentage of freezing behavior in training context A and similar context B. Note that LIN28A WT and cKO mice showed comparable freezing behavior in both context A and B. Repeated measures ANOVA was performed. Context A: P = 0.247, F(1,25) =

1.407. Context B: $P = 0.338$, $F(1,25) = 0.954$. WT (n = 9), cKO (n = 18). **(D)** A graph showing discrimination ratio with no change between LIN28A WT and cKO mice. Repeated measures ANOVA was performed. $P = 0.661$, $F(1,25) = 0.197$. WT (n = 9), cKO (n = 18). **(E)** A graph showing the freezing percentage when sham-manipulated LIN28A WT and cKO mice were exposed to training context A, similar context B, and completely new context C. Note that both LIN28A WT and cKO mice could recognize context B and C as different contexts compared to original context A. Student's paired t-test was performed. LIN28A WT: context A vs. B, $P = 0.011$, $t(8) = 3.288$; context B vs. C, $P = 0.092$, $t(8) = 1.910$; context A vs. C, $P = 0.034$, $t(8) = 32.543$. LIN28A cKO: context A vs. B, $P < 0.001$, $t(17) = 4.619$; context B vs. C, $P = 0.090$, $t(17) = 1.795$; context A vs. C, $P = 0.002$, $t(17) = 3.686$. WT (n = 9), cKO (n = 18). Data are presented as mean ± SEM. $^*P < 0.05$. ns, not significant.
